# Self-assembly of MPG1, a hydrophobin protein from the rice blast fungus that forms functional amyloid coatings, occurs by a surface-driven mechanism

**DOI:** 10.1038/srep25288

**Published:** 2016-05-04

**Authors:** Chi L. L. Pham, Anthony Rey, Victor Lo, Margaux Soulès, Qin Ren, Georg Meisl, Tuomas P. J. Knowles, Ann H. Kwan, Margaret Sunde

**Affiliations:** 1 Discipline of Pharmacology, School of Medical Sciences, University of Sydney, NSW 2006, Australia; 2 School of Life and Environmental Sciences, University of Sydney, NSW 2006, Australia; 3 Department of Chemistry, University of Cambridge, Cambridge CB2 1EW, United Kingdom

## Abstract

Rice blast is a devastating disease of rice caused by the fungus *Magnaporthe oryzae* and can result in loss of a third of the annual global rice harvest. Two hydrophobin proteins, MPG1 and MHP1, are highly expressed during rice blast infections. These hydrophobins have been suggested to facilitate fungal spore adhesion and to direct the action of the enzyme cutinase 2, resulting in penetration of the plant host. Therefore a mechanistic understanding of the self-assembly properties of these hydrophobins and their interaction with cutinase 2 is crucial for the development of novel antifungals. Here we report details of a study of the structure, assembly and interactions of these proteins. We demonstrate that, *in vitro*, MPG1 assembles spontaneously into amyloid structures while MHP1 forms a non-fibrillar film. The assembly of MPG1 only occurs at a hydrophobic:hydrophilic interface and can be modulated by MHP1 and other factors. We further show that MPG1 assemblies can much more effectively retain cutinase 2 activity on a surface after co-incubation and extensive washing compared with other protein coatings. The assembly and interactions of MPG1 and MHP1 at hydrophobic surfaces thereby provide the basis for a possible mechanism by which the fungus can develop appropriately at the infection interface.

Rice is the staple food for half of the global population. Infection by the rice blast fungus, *Magnaporthe oryzae*, is responsible for loss of up to one third of the annual global rice harvest, an amount that could feed one billion people^1^. *M. oryzae* also infects food grasses and new strains of the fungus that infect wheat have recently emerged in South America^2^. Notwithstanding environmental challenges, rice crop productivity must be increased in the face of a growing global population. *M. oryzae* rapidly overcomes blast resistance and cultivars become ineffective in 2–3 years^2^, so alternative strategies are urgently needed to curb the infectivity of *M. oryzae* and protect food security.

Members of the fungal hydrophobin and cutinase protein families have been demonstrated to be centrally involved in infection of rice by *M. oryzae*^3^,^4^,^5^,^6^. Hydrophobins are small surface-active proteins that can reduce the surface tension of an aqueous growth medium to facilitate production of aerial hyphae and spores. Additionally, they coat the surface of the spores to reduce wettability and facilitate surface interactions^7^. Class I hydrophobins self-assemble to form functional amyloid structures known as rodlets which associate laterally to form amphipathic, fibrillar layers. Class II hydrophobins form amphipathic protein layers that lack the fibrillar structure. Fungal cutinases are able to degrade cutin, the waxy polymer that coats plant aerial structures, and a wide variety of other biopolymers. These enzymes play a critical role in recycling of plant material in addition to being important for pathogenesis^8^. Rice plant infection by *M. oryzae* requires secreted cutinase activity to break down the cutin covering on the rice leaf surface and release cutin monomers. These cutin monomers stimulate the cAMP/PKA and DAG/PKC-mediated signal transduction cascades that lead to development of the specialised infection structure known as the appressorium and subsequent penetration of the plant tissue^3^,^5^,^6^,^9^. In addition to cutinase activity, *M. oryzae* requires the presence of a hydrophobic surface signal for normal development of the infection structures^1^. Talbot and colleagues suggested that rice blast hydrophobins might act as sensors of the hydrophobic surface of the leaf and trigger development of appressoria from spores^5^.

The spores of *M. oryzae* are covered with a layer composed of the hydrophobin MPG1 and the protein is also present in appressoria^4^. When leaves are showing blast lesions, MPG1 represents half of the expressed transcripts^10^. Knockout of MPG1 results in impaired appressorium development and reduced infectivity^4^. The genome of *M. oryzae* also encodes MHP1, another hydrophobin that appears to share overlapping but non-redundant activities with MPG1 and four other putative hydrophobins^11^. *M. oryzae* produces multiple cutinases and cutinase 2 (Cut2) has been demonstrated to be necessary for *M. oryzae* to sense a hydrophobic surface and initiate differentiation, penetration and full virulence^1^.

In order to elucidate the assembly properties of the hydrophobins and the interplay between them and Cut2 at the interface associated with rice blast infection, we have characterised the structures and assemblies formed from MPG1 and MHP1 as well as the activities of Cut2 from *M. oryzae* in the presence of MPG1 and MHP1. The structure of the monomeric form of MPG1 in solution shares the amphipathic distribution of charged and non-charged surface areas and the core β-sheet structure observed in other hydrophobins. We observe fast-timescale dynamics of two inter-cysteine segments in the protein that might foreshadow the conformational change that occurs when this protein self-assembles into amyloid-like fibrillar structures. In contrast, the hydrophobin MHP1 displays behaviour consistent with its characterisation as a class II hydrophobin and while no specific protein:protein interactions can be detected between these two hydrophobins, MHP1 can inhibit MPG1 self-assembly. We observe that the hydrolytic activity of Cut2 is stimulated by interaction with folded polypeptides in a manner that displays little sequence specificity and the enzyme binds to protein-coated surfaces, with increased Cut2 activity detected on a surface coated with the amyloid rodlet form of MPG1. We show that MPG1 self-assembly into functional amyloid rodlets is strictly limited to a hydrophobic:hydrophilic interface. This surface-driven mechanism potentially provides the means for recognition of an appropriate infective surface, with subsequent triggering of cutin monomer generation. This new understanding of the mechanism of MPG1 assembly and of interactions between MPG1, MHP1 and Cut2 may lead to identification of targets for disruption of rice blast infection.

## Results

### Solution structure and dynamics of MPG1

**Figure 1:**
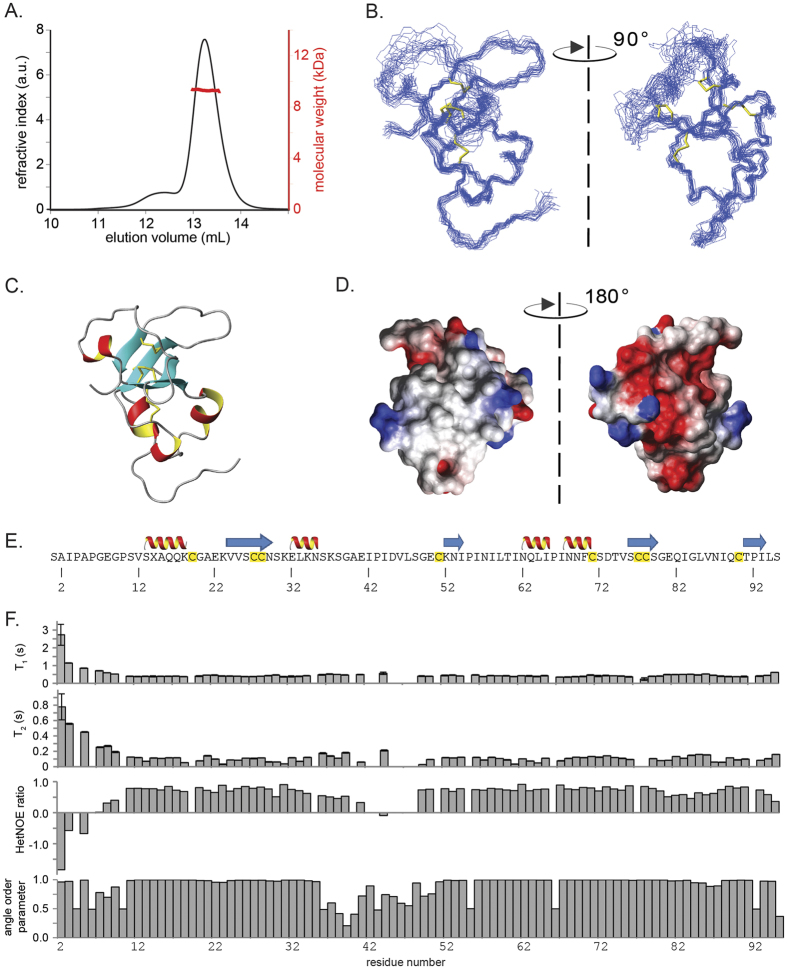
Structure and dynamics of MPG1 in solution. (**A**) SEC-MALLS elution profile of purified MPG1. (**B**) Overlay of the 20 lowest energy conformers of MPG1, with the four disulphide bonds shown in yellow. (**C**) Ribbon representation of secondary structure of MPG1. (**D**) Surface-charge distribution of MPG1 showing the amphipathic nature of MPG1, with charges coloured in red and blue and hydrophobic regions in white. (**E**) Distribution of secondary structure elements along the primary sequence of MPG1. (**F**) Plots of ^15^N *T*_1_, *T*_2_, Heteronuclear NOE values and angle order parameter *vs.* residue number.

MPG1 was produced recombinantly in *E. coli* as previously described^12^,^13^. Under gel filtration chromatography a single main peak was observed, with in-line multi-angle laser-light scattering (MALLS) measurements indicating that MPG1 is a monomer in solution at a concentration of ~30 μM as the protein eluted from gel filtration chromatography (experimental mass 9.3 ± 0.9 kDa *vs*. theoretical mass 9.9 kDa; Fig. 1A). One-dimensional ^1^H and two-dimensional ^1^H-^15^ N-HSQC (Heteronuclear Single Quantum Coherence) spectra were initially recorded on samples of MPG1 at concentrations ranging from 40 to 100 μM for condition screening to obtain the best quality NMR spectra^13^. No change to peak positions or relative intensities was observed as the protein concentration was subsequently increased to ~400 μM for collection of NMR spectra used for structure determination, indicating that MPG1 maintains its fold and the protein does not undergo self-association over the concentration range 40 to 400 μM.

The three-dimensional structure of the MPG1 monomer was then determined using solution-state NMR spectroscopy. A single set of resonances was observed for all residues except for A2, I3, S11, and T74 (all with two sets of peaks) and A5, E8, G39, and A40 (all with three sets of peaks)^13^. The multiple peak sets occur mainly for residues located in the N-terminus and suggest conformational flexibility in this region. Where more than one set of resonances has been identified, structure calculations were performed using peaks belonging to the most intense set. The family of calculated structures exhibits 99.9% of the structured residues in allowed regions of the Ramachandran plot and the 20 conformers with the lowest energy are shown in Fig. 1B.

The MPG1 structure is centred on a four-stranded curved β-sheet which forms an open barrel (strands comprised of residues V24–C28, C51–N53, V75–C78, and Q89–C90), surrounded by four α-helices (formed by residues V12–K18, K31–N35, P55–L64, and P66–N69) (Fig. 1C). The structure of MPG1, with many different elements of regular secondary structure and an extended flexible region outside the β-sheet core, is consistent with its classification as a class I hydrophobin^14^. The disulphide-bonding pattern in the MPG1 structure is well supported by NOE data and is the same as in all other hydrophobin structures determined to date^15^. Two of the four disulphide bonds (C27-C51 and C78-C90) lie in the barrel, with each linking two strands, whereas the other two (C26-C71 and C19-C77) connect the outside surface of the barrel to the α-helices. Over the structured regions A2–N35 and C51–L94 (the residues with Φ and ψ-angle order parameters >0.9), the 20 lowest energy structures display a backbone r.m.s.d. of 0.71 Å.

The structure of MPG1 displays a separation of charged and uncharged residues on opposing surfaces, a distribution also observed on other hydrophobins^12^,^16^ (Fig. 1D). The two aspartates, six glutamates and six lysines are largely clustered on one half of the protein surface, except for Glu7 which is located near the relatively disordered N-terminus, and Lys17 which is positioned on the boundary of the charged and uncharged areas. The adjacent Glu21 and Lys22 residues appear to form a salt bridge. This segregation of charged and uncharged regions on the surface is consistent with the high surface activity displayed by hydrophobin proteins^17^.

^15^N-*T*_1_ and *T*_2_ relaxation and hetero-NOE experiments indicate that the N-terminus of MPG1 is flexible in solution. The remainder of the MPG1 structure is relatively well-ordered, except for the large ~16 residue flexible region that follows the short helix in the Cys3-Cys4 loop (Fig. 1E,F). This region is characterised by a lack of medium- and long-range NOEs and displays a substantial decrease in hetero-NOE ratios. The MPG1 structure has been deposited into the Protein Data Bank with accession code 2N4O.

### Comparison of the structures of MPG1 and MHP1 with other hydrophobins

**Figure 2:**
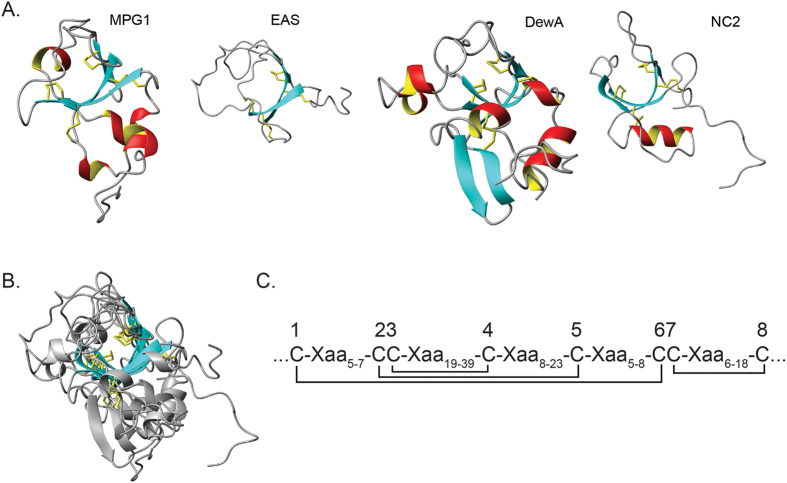
Hydrophobins adopt an open β-barrel structure in solution. (**A**) Solution structures of MPG1 and other Class I hydrophobins (EAS and DewA) and a Class II hydrophobin (NC2). (**B**) An overlay of hydrophobin structures based on the core β-strands illustrates the common half β-barrel structure. (**C**) Schematic representation of the general sequence features of hydrophobins, in particular the unique pattern of eight cysteine residues and four disulphide bonds.

Comparison of the structures of MPG1 and other class I hydrophobins reveals a high level of structural similarity, particularly with EAS^12^ from *Neurospora crassa* (Fig. 2A). In MPG1 and EAS, a similar curved β-sheet structure is formed by the β-strands and residues around Cys3, Cys4, Cys7 and Cys8. However in the EAS monomer structure, contacts (including two hydrogen bonds) between the Cys7-Cys8 segment and the rest of the β-sheet core are observed, leading to a more closed barrel conformation^12^. The structures of MPG1 and DewA^16^, a class I hydrophobin from *Aspergillus nidulans*, both display many secondary structural elements outside the β-barrel core. However most of these, apart from the helical region near the N-terminus, are not conserved (Fig. 2A). An overlay of hydrophobin structures, based on the core β-strands of the half-barrel, illustrates the manner in which the hydrophobin fold accommodates the many different sequence variations and structural features found in Class I fungal hydrophobins (Fig. 2B,C).

MHP1 is also involved in *M. oryzae* morphogenesis and plant colonization^11^. In contrast to MPG1, however, MHP1 shares 78% sequence identity and the same inter-cysteine spacing as NC2, a class II hydrophobin from *N. crassa* for which we have previously solved the solution structure^18^. The homology model of MHP1 based on NC2 indicates that it is likely to adopt the open barrel structure observed in the solution structure of NC2 (Fig. 2A).

### MPG1 and MHP1 are surface active and form amphipathic films

**Figure 3:**
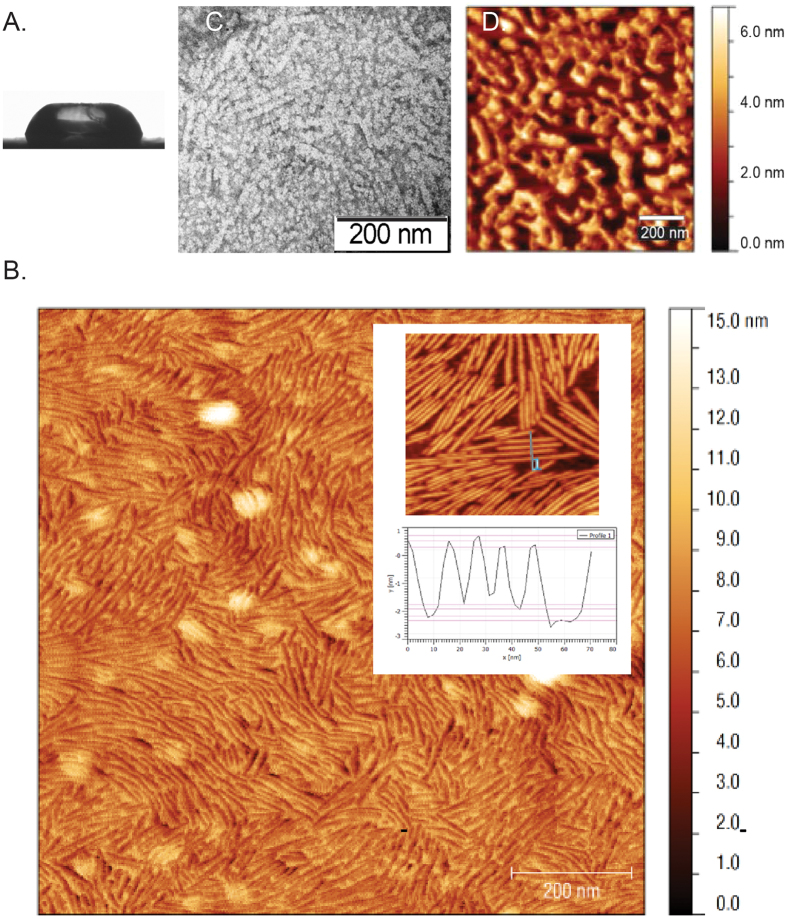
Morphology of self-assembled MPG1 and MHP1. (**A**) Image of a droplet of MHP1 on a hydrophobic OTS-coated silicon surface. (**B**) AFM image of MPG1 on HOPG with the inset showing a height profile across MPG1 rodlets as indicated. (**C**) Negatively stained TEM image of MPG1 rodlets assembled on TEM grid. (**D**) AFM image of MHP1 on HOPG.

The proposed roles for hydrophobins on the waxy rice leaf led us to assess the structures and properties of MPG1 and MHP1 layers formed at interfaces. Initially the surface activity of the soluble forms of the hydrophobins was assessed. Droplets of purified MPG1 and MHP1 solutions placed onto a hydrophobic OTS-coated silicon surface displayed significantly reduced contact angles (58° ± 10° and ~60° for MPG1 and MHP1, respectively) compared with water droplets alone (94° ± 3°) or droplets containing lysozyme, which has a similar molecular mass, at the same concentration (79° ± 7°). Only one of the three MHP1-containing drops displayed a shape profile that could be fitted to determine a value for the contact angle, with the other two displaying an angular and irregular drop shape, suggesting MHP1 forms a discrete protein layer on the surface of the droplets (Fig. 3A). The distortion of drop shape has been observed for many other hydrophobins, including for NC2 and DewA^16^,^18^. The reduction in contact angle observed with MPG1 and MHP1 and the irregular drop shape observed for solutions of MHP1 indicates they are highly surface active proteins.

Subsequently, samples of purified MPG1 and MHP1 solutions were allowed to dry overnight at room temperature onto a freshly-cleaved highly oriented pyrolytic graphite (HOPG) surface and the morphology of the layers was examined by atomic force microscopy (AFM). MPG1 spontaneously assembled into rodlet structures that formed a lateral film across the surface (Fig. 3B). This protein film had a height of 2.5 ± 0.2 nm (measured from an area of the sample where bare HOPG was visible between the rodlets, Fig. 3B inset). These MPG1 assemblies could also be visualised by negative-stain TEM on a carbon-coated formvar film supported by a copper grid. A densely-packed covering of protein was observed, comprising of rodlets of ~200 nm in length and ~20 nm in width (Fig. 3C), consistent with the morphology observed by AFM. In contrast, while a patterned protein layer with a height profile ranging from 0 to ~5 nm was observed for MHP1 on HOPG by AFM, MHP1 layers do not display the defined rodlet or polygonal morphology typically observed for Class I and II hydrophobin assemblies, respectively (Fig. 3D)^14^. Water contact angles were measured on areas coated with dried down MPG1 and MHP1 on Octadecyltrichlorosilane (OTS)-coated silicon. Both protein layers significantly increased the wettability of this surface as shown by a reduction of water contact angles from ~100° to ~62° and ~57°, respectively, indicating that MPG1 and MHP1 both spontaneously form amphipathic films at interfaces. These values compared well with water contact angles measured for coatings formed from other hydrophobins, which ranged from ~49° for NC2 (a class II hydrophobin from *Neurospora crassa*) to ~69° for EAS_Δ15_ (a modified class I hydrophobin from *Neurospora crassa*)^14^,^18^.

### MPG1 but not MHP1 assemblies are amyloid-like

**Figure 4:**
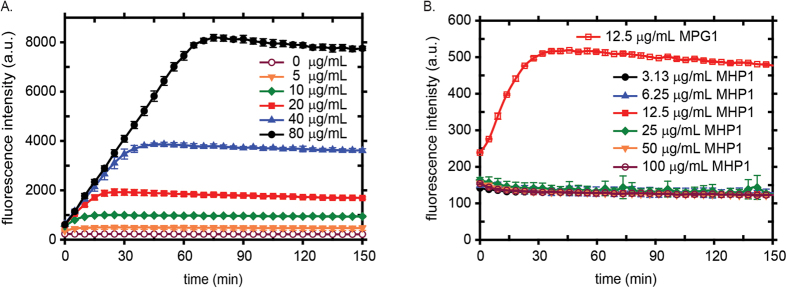
MPG1 but not MHP1, assembles into amyloid-like rodlets. (**A**) *In vitro* agitation of MPG1 solutions at pH 7.2 over protein concentration range of 0–80 μg/mL. (**B**) No increase in ThT fluorescence is observed when MHP1 solutions up to 100 μg/mL are incubated with agitation.

Thioflavin-T (ThT; an amyloid-specific dye) binding assays were performed over a range of MPG1 and MHP1 concentrations and at different temperatures to determine whether MPG1 and MHP1 assemblies are built on an underlying amyloid substructure. These assays involve vigorous agitation of ThT-containing protein solutions in a fluorescence plate reader. We have previously demonstrated that exposure to hydrophobic:hydrophilic interfaces at the solution surface and on internal air bubbles, with continuous regeneration of interfaces by agitation, causes class I hydrophobins to self-assemble into rodlets^14^,^16^. Consistent with published data for other class I hydrophobins^16^,^19^, MPG1 assemblies readily bind ThT and give rise to fluorescence at 485 nm, indicating the presence of an amyloid substructure. The initial rate of MPG1 assembly is rapid, does not show a lag phase and is relatively insensitive to protein concentration (Fig. 4A). A linear increase in ThT fluorescence was observed for most of the time course with the final fluorescence intensity being proportional to the protein concentration. In contrast to the situation for DewA assembly^16^, higher MPG1 protein concentrations do not appear to inhibit rodlet assembly.

In contrast, MHP1-ThT mixtures did not develop significant levels of fluorescence, even following extended periods of agitation at MHP1 concentrations as high as 100 μg/mL (Fig. 4B). This result is consistent with both the sequence of MHP1 and the non-fibrillar nature of the MHP1 layers we observed by AFM. Together, these data confirm that MHP1 is a Class II hydrophobin that does not possess amyloidogenic characteristics.

### Hydrophobins and other proteins enhance the activity of Cut2 from *M. oryzae*

**Figure 5:**
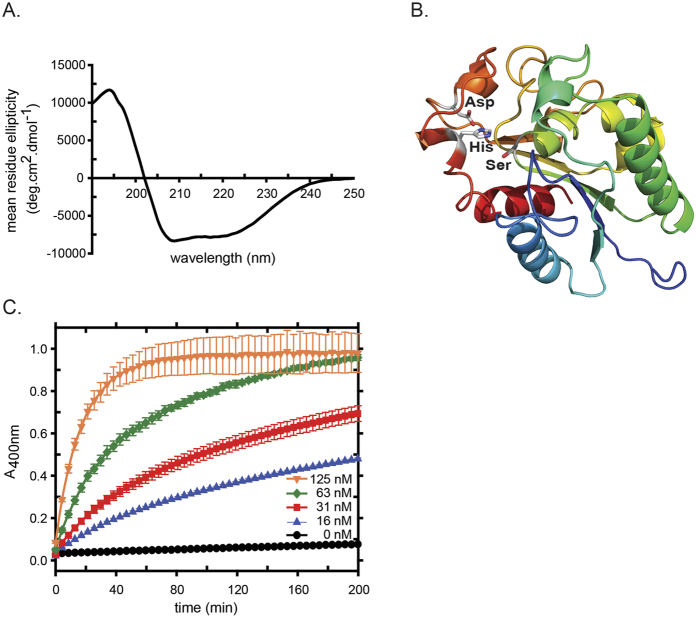
Recombinant expression and purification of Cut2 yield a fully folded and functional enzyme. (**A**) Far-UV CD spectrum from purified recombinant Cut2 indicates a predominantly α-helical structure. (**B**) Structural homology model of *M. oryzae* Cut2, based on the structure of Cut1 from *F. solani*. (**C**) Recombinant Cut2 is active as indicated by the increase in esterase activity upon addition of increasing enzyme concentrations.

Skamnioti and Gurr^3^ have postulated that one or more of the hydrophobins from *M. oryzae* may be adsorbed onto the leaf surface and subsequently recruit fungal cutinases to facilitate cutin degradation and penetration of the plant host. *M. oryzae* Cut2 is thought to be the most likely candidate cutinase^1^. We prepared recombinant *M. oryzae* Cut2 and characterised its structure and activity. The recombinant Cut2 is folded and gives rise to a far-UV circular dichroism spectrum that is consistent with a homology model based on residues 35–214 of the structure of *Fusarium solani* cutinase, which shares a sequence identity with *M*. *oryzae* Cut2 of ~38% (Fig. 5A,B). The predicted structure of *M. oryzae* Cut2 has an α/β fold, with five parallel β strands at the centre of the enzyme forming a sheet flanked by eight α-helices and with the catalytic triad (Asp, His, Ser) exposed to the solvent (Fig. 5B). We determined *k*_cat_/*K*_*m*_ for our recombinant Cut2 with *p*-nitrophenylbutyrate as substrate (Fig. 5C). The value of 0.20 μM^−1^min^−1^ is similar to the values of 0.26 ± 0.06 μM^−1^min^−1^ and 3.49 ± 0.51 μM^−1^min^−1^ reported for *F. solani* and *A. oryzae* cutinases, respectively, acting on this substrate^20^.

**Figure 6:**
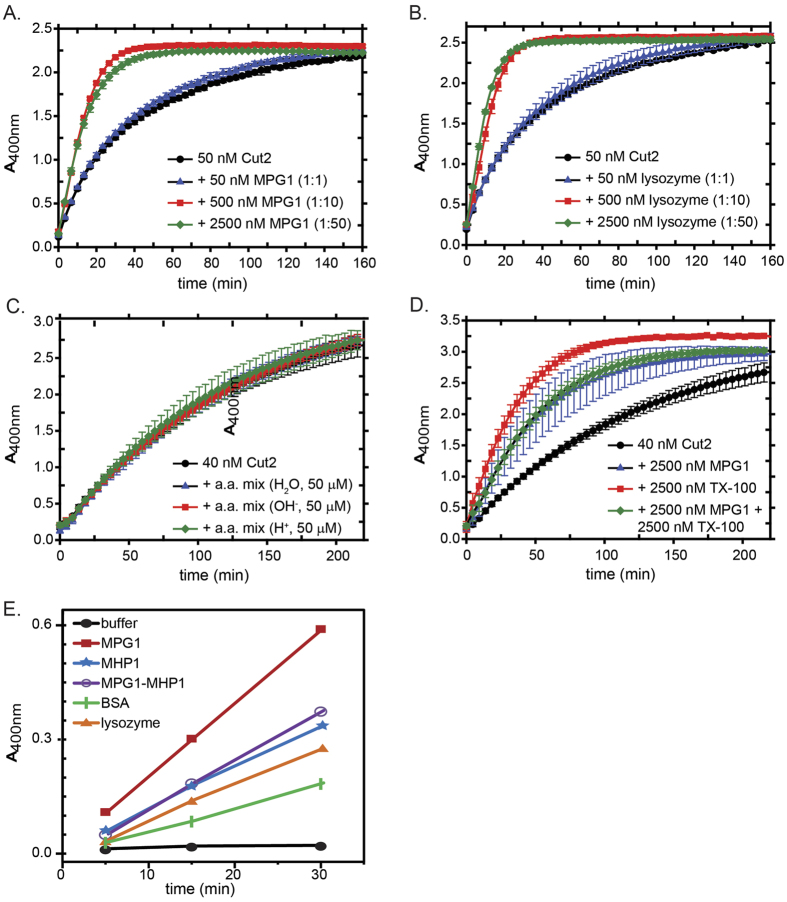
Activation of Cut2 enzymatic activity by proteins and detergents. (**A**,**B**) Effect of MPG1 and lysozyme in molar excess on the esterase activity of Cut2. (**C**) Addition of mixtures composed of single amino acids has no effect on the activity of Cut2. (**D**) Effect of the detergent Triton X-100 on the activity of Cut2. (**E**) Recruitment and activation of Cut2 by proteins dried onto a HOPG surface.

In the presence of increasing amounts of MPG1 and MHP1 in solution, a marked enhancement of Cut2 activity was observed when the hydrophobins were in excess (Fig. 6A). However, lysozyme was observed to produce a similar increase in the substrate hydrolysis rate by Cut2 (Fig. 6B). Mixtures of single amino acids or short peptides had no effect on Cut2 activity but addition of the detergent Triton^TM^ X-100 resulted in an increase in Cut2 activity (Fig. 6C,D), in contrast to the observations of Sehgal *et al.*^20^ and Brissos *et al.*^21^ who demonstrated that *F. solani* cutinases are destabilised by detergents^21^,^22^. The level of enhancement by the detergent is similar to that seen with excess protein; however, combined addition of Triton^TM^ X-100 and MPG1 did not produce an additive effect, suggesting a saturable interaction with other macromolecules. These results indicate that Cut2 activity is stimulated by interaction with other macromolecules in solution but that the interaction is not specific. Unlike typical lipases, the activity of fungal cutinases has not been reported to be stimulated by interfaces, a characteristic attributed to the absence of a hydrophobic loop over the active site^8^ so the molecular basis for this activation remains unclear.

Takahashi and co-workers^23^ have reported that binding of the *Aspergillus oryzae* hydrophobin RolA to the *A. oryzae* cutinase CutL1 stimulates degradation of the biodegradable polyester polybutylene succinate-coadipate by CutL1. The recruitment of CutL1 only occurs to the assembled form of RolA on a surface and is modulated through ionic interactions between charged residues on the hydrophobin and the cutinase^24^. We compared the binding and activity of Cut2 after incubation of the cutinase in contact with a range of proteins deposited onto a HOPG surface under conditions where we had previously observed the formation of MPG1 rodlets by AFM (Fig. 6E). Extensive washing of the protein-coated HOPG surfaces was carried out after the incubation with Cut2 to remove non-bound and loosely bound protein before the substrate was added and its conversion to product measured. Only negligible signal was observed with a bare HOPG surface suggesting that Cut2 binds minimally to bare HOPG, a highly hydrophobic surface, or is not active after binding to bare HOPG. In contrast, layers of deposited proteins including MHP1, a 50:50 mixture of MPG1 and MHP1, BSA and lysozyme were able to recruit Cut2 in an active form. The highest signal was observed with the MPG1-coated HOPG surface, suggesting a substantial binding and/or activation of Cut2 by the MPG1 rodlet layer. The layers of mixed MPG1 and MHP1 and MHP1 alone were observed to recruit or activate Cut2 more than lysozyme or BSA but these results suggest that binding of *M. oryzae* Cut2 to a surface is not protein-specific. Neither does it appear to be mediated by overall protein charge and ionic interactions: MPG1 (pI 4.9), MHP1 (pI 3.9), BSA (pI 4.7) and Cut2 (pI 6.7) are predicted to be negatively charged under the assay conditions used while lysozyme (pI 11.4) is positively charged. However, the level of activity of cutinase activity that we observe upon binding to a protein-coated follows this series: MPG1>MHP1>lysozyme>BSA. In support of this, Kershaw and coworkers have shown that other hydrophobins can substitute effectively for MPG1 when they are expressed under control of the MPG1 promoter and observed to form rodlets on the conidial surface^25^. While the interaction between Cut2 and the MPG1 rodlet layer does not appear to be highly specific, our results show that the MPG1 rodlet coating on the hydrophobic surface provides an effective binding surface for Cut2 that is compatible with cutinase catalytic activity.

### MPG1 assembly is modulated by other proteins, including MHP1 and other surface active agents

**Figure 7:**
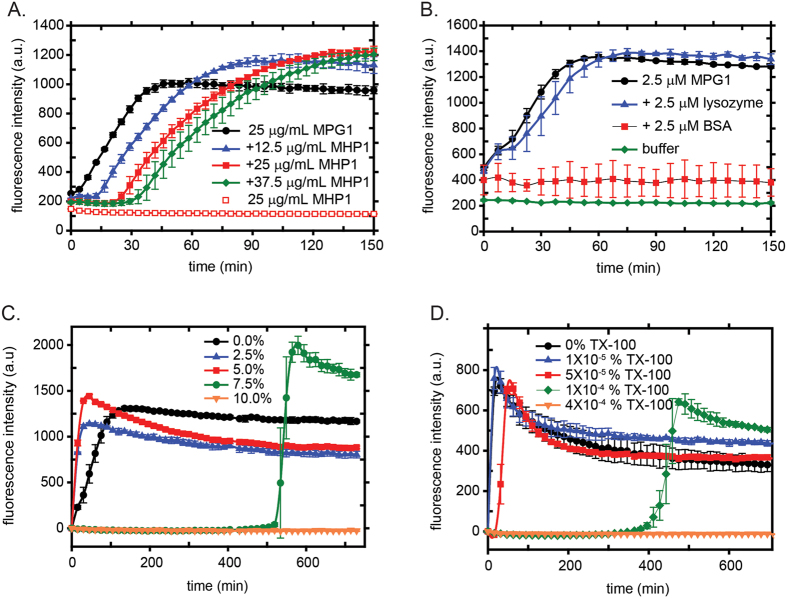
MPG1 rodlet formation is modulated by the presence of other proteins and surface active agents. (**A**) Dose-dependent introduction of a lag phase in MPG1 assembly when the Class II hydrophobin MHP1 is co-incubated with MPG1. (**B**) Assembly of MPG1 in the presence of the non-surface-active protein lysozyme and the surface active protein BSA at stoichiometric concentrations. (**C,D**) Reductions in the surface tension by addition of alcohols such as isopropanol (panel **C**) or a detergent such as Triton X-100 (panel **D**) result in introduction of a lag phase, followed by a rapid elongation.

We next investigated the effect of MHP1 on MPG1 assembly, given that both proteins are surface-active and highly expressed during *M. oryzae* infection. The presence of MHP1 introduces a lag phase to MPG1 rodlet assembly (Fig. 7A) in a dose-dependent manner, with the length of the lag phase increasing at higher concentrations of MHP1. We tested whether stoichiometric amounts of lysozyme and bovine serum albumin (BSA) affect MPG1 assembly. Lysozyme is a small protein and has a similar mass to MHP1 but is not surface active whereas BSA is a large, surface-active protein and has been used in applications and formulations where high surface activity is required^26^. As shown in Fig. 7B, the rate of MPG1 rodlet assembly is not affected by lysozyme but the presence of BSA completely inhibited MPG1 assembly over the time-scale of the experiment. Lysozyme and BSA do not form ThT-positive assemblies on their own under the experimental conditions used. These results suggest that the MPG1 assembly is sensitive to the presence of other surface active proteins, including MHP1. However, no direct interaction could be detected between biotinylated MPG1 immobilised onto a streptavidin biosensor and MHP1 in solution using bio-layer interferometry and no changes to either peak positions or intensities were observed in ^1^H-^15^N-HSQC NMR spectra when unlabelled MHP1 was titrated into ^15^N-labelled MPG1.

We have previously shown that surface tension plays a critical role in controlling the assembly of other hydrophobins^27^. We therefore investigated the effect of surface tension on MPG1 self-assembly to form amyloid rodlets. Reduction of the surface tension through the addition of alcohols or detergents resulted in observation of a lag phase before rapid MPG1 rodlet assembly. When the surface tension was reduced below ~40 mN/m, by addition of an alcohol (Fig. 7C) or a detergent (Fig. 7D), no rodlet assembly was detected even after 10 hours of agitation. As observed previously with the hydrophobins EAS and DewA, the nature of the additives was not the determining factor, with alcohols and detergents producing a similar effect^27^. Instead, the surface tension of the solution appears to control or limit the initiation of rodlet assembly. Therefore, if MHP1 does modulate the rate of MPG1 assembly in the rice blast fungus, this is likely due to its effect on surface tension or on a surface, rather than to a specific protein:protein interaction in solution.

### A model for interface-controlled MPG1 assembly

**Figure 8:**
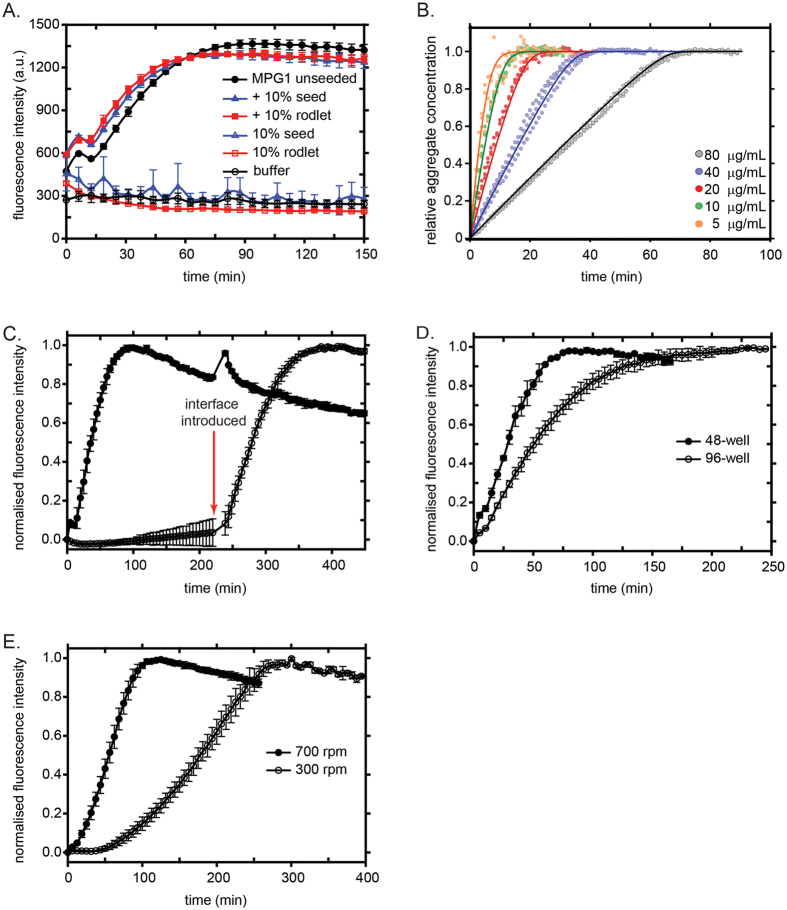
The hydrophobic:hydrophilic interface is essential for rodlet formation by MPG1. (**A**) Addition of pre-formed rodlets or seeds has no effect on rodlet formation by MPG1. (**B**) Fit of “dock-lock” kinetic mechanism to the normalised aggregation data presented in Fig. 4A. (**C**) In the absence of an interface, no assembly is observed (open circles) while upon introduction of an interface (arrow), assembly is initiated and parallels that observed when an interface is present from the start of assay (closed circles). (**D**) Rate of elongation increases with increase in surface area. (**E**) The rate of self-assembly is positively correlated with the rate of agitation of the solutions.

MPG1 self-assembly clearly displays a kinetic profile that is very different to the concentration-dependent sigmoidal curves commonly observed for other amyloidogenic peptides and proteins^28^. Two notable features are apparent: the slope is independent of the monomer concentration and the curves of total aggregate concentration versus time increase linearly and then plateau (Fig. 4A). These observations yield two constraints on the possible mechanisms of MPG1 assembly: (1) The fact that the initial rate is the same for different monomer concentrations indicates that the rate determining step is monomer independent until monomer is significantly depleted, that is, when the reaction is about to plateau, and (2) the linear increase in ThT fluorescence suggests that the number of elongating aggregates remains constant with time. This situation contrasts with the aggregation profiles observed for most other amyloidogenic peptides and proteins, where the number of growth-competent aggregates increases with time, as nucleation processes continuously produce new aggregates, resulting in a positive curvature for ThT intensity plots at early timepoints^29^,^30^. The constant slope for the MPG1 aggregation reaction observed here indicates that the rate of growth at any given point is determined by a constant number of growing aggregates. That is, although the total amount of aggregates increases during the course of assembly, the amount of growth-competent aggregates is somehow limited and remains constant. This property is also expected to significantly affect the system’s response to seeding. In aggregation reactions that take place in solution, adding preformed seeds at the start of the experiment has significant effects on the kinetics, by providing more growth competent fibrils and thereby speeding up aggregation. For our proposed surface controlled mechanism however, the maximal number of growth-competent fibrils is limited (possibly by the size of the interface) and is reached effectively immediately after the start of the reaction. Therefore this scheme predicts that adding preformed rodlets or seeds would have no detectable effect on MPG1 assembly. In line with this prediction, the addition of rodlet fragments had no effect on MPG1 rodlet formation (Fig. 8A) in contrast to simple nucleated polymerisation where addition of pre-formed aggregates reduces the lag phase^31^.

Based on our observations, a model for the assembly of MPG1 amyloid rodlets can be proposed. Initially, nucleation must occur to convert soluble monomers to small aggregates. This process is too fast to be observed but proceeds for only a short period before the limiting concentration of growth competent aggregates is reached. These aggregates can elongate by addition of monomers to their ends. Addition occurs in a Michaelis-Menten like process consisting of an initial monomer attachment step that is dependent on monomer concentration, followed by a monomer-concentration-independent rearrangement step as the monomer locks into the aggregate^32^,^33^. This kind of “dock-lock” mechanism has been described in the aggregation of other proteins under specific conditions^34^ and leads to two predictions. First, for high monomer concentrations, the rearrangement step is rate-limiting and the rate of growth does not depend on the monomer concentration. Second, for low monomer concentrations, the monomer concentration-dependent attachment step *is* rate-limiting and the elongation process becomes monomer-dependent. The equations that describe such a scheme are given in the Methods. To assess the plausibility of this assembly model we constructed the corresponding rate equations and fitted them globally to the normalised aggregation data (Fig. 8B). Our entire data set fits the model extremely well with just two parameters, namely the elongation rate constant and the Michaelis constant of elongation, suggesting that our model is consistent with the observed MPG1 assembly process.

One key question that arises from this model is: “What is the mechanism underlying the restriction of nucleation events and the consequent independence of assembly kinetics to monomer concentration?” Consistent with the high surface activity of hydrophobins, we propose that the available interface area provides the key. If aggregates can grow only when they are adsorbed at the air:liquid interface, then the number of growth-competent aggregates is limited by interface area and remains constant once the interface is saturated with protein.

To test these ideas, we manipulated the size of the interface. First, the interface was removed by filling the wells completely, with exclusion of all air between the liquid and the sealing film. The absence of a hydrophobic:hydrophilic interface, even with extensive agitation, completely prevented self-assembly of MPG1 into ThT-positive rodlets (Fig. 8C). However, once the interface was reintroduced through removal of a small amount of the total sample volume from the well, rodlet formation was immediately initiated. Under these conditions, the curve resembled that observed when the interface was available from the start, suggesting that the air:liquid interface is critical and is the limiting factor for rodlet assembly. This mechanism also predicts that an increase in the surface area under conditions of constant volume should lead to an increase in the rate of rodlet assembly. We therefore performed the assay in larger wells while keeping the volume constant and a more rapid rate of formation was observed, consistent with our model (Fig. 8D). Furthermore, when the MPG1 assembly assay is performed at a slower agitation rate (and therefore a slower turnover of the air:liquid interface), a significantly reduced rate of assembly is observed (Fig. 8E). These results indicate that rodlet nucleation and elongation only occur at the interface.

## Discussion

**Figure 9:**
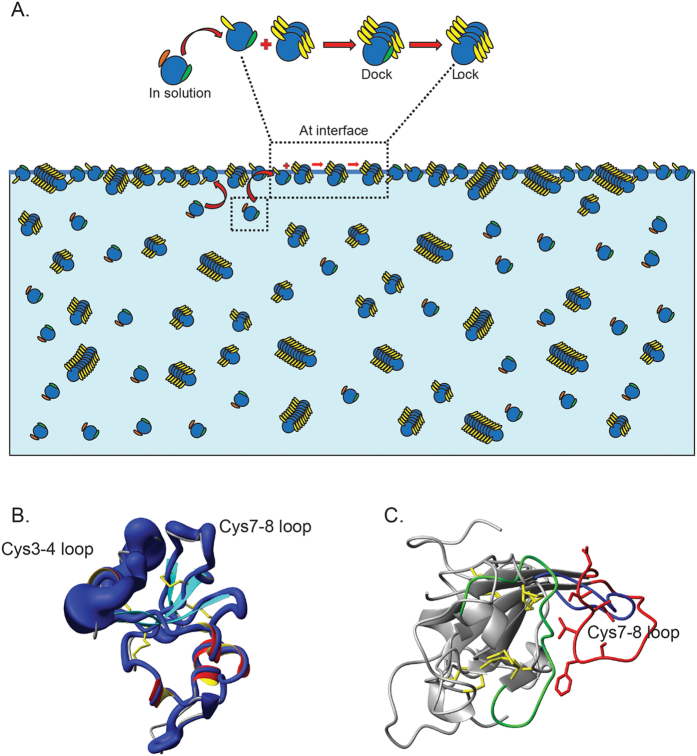
Schematic model of MPG1 assembly and the potential role played by the Cys7-Cys8 loop. (**A**) Conformational change to an assembly-competent form of MPG1 is limited to the interface. Elongation occurs via a two-step process that involves docking of monomer to the growing rodlet and locking into a rodlet form. (**B**) Ribbon diagram of MPG1 is overlaid with a sausage-style cartoon representation of the MPG1 structure with the diameter of the tubular spline corresponding to the flexibility experienced by the backbone atoms. (**C**) Overlay showing the position of the Cys7-Cys8 loop in the MPG1 structure (blue) in solution and that of EAS in solution (green) and in the EAS rodlet model (red).

This is the first time that the assembly kinetics of a hydrophobin and a functional amyloid have been described mathematically. The kinetics of MPG1 assembly can be fitted to a Michaelis-Menten-like dock-lock process. Although this simple model consisting of an attachment stage and a conformational rearrangement step fully describes the observed kinetics, the molecular interpretation of these kinetics is less obvious. A saturating elongation rate has been observed for many other amyloids and is commonly rationalised by considering the requirement for proteins to adopt the correct β-sheet structure when locking into the amyloid state, an easily rate-limiting process when the initial attachment of proteins to the fibril ends is fast. Alternatively, the surface may be saturated in monomers already at low bulk monomer concentrations and if only surface-bound species can elongate, then the monomer concentration-independence of the elongation process can simply be a result of the number of surface-bound monomers being saturated and hence independent of the bulk monomer concentration. The same kinetic equations still apply provided that the first, monomer-dependent step is interpreted as the binding to the surface rather than to the fibril ends. In contrast, the observation of a constant number of growth-competent fibrils throughout a reaction across different monomer concentrations and a range of experimental conditions is rare; this feature might well be unique to hydrophobin assembly with its strict requirement for a hydrophobic:hydrophilic interface (Fig. 9A). By linking the number of growth-competent aggregates to the available interface and not to protein concentration, a specific mechanism exists for the fungus to have a pool of soluble hydrophobin molecules that can assemble as soon as the right environment is presented but not before. Given that erroneous and unintentional formation of amyloids often has serious biological consequences^35^, it is perhaps unsurprising that hydrophobins have evolved to only assemble at hydrophobic:hydrophilic interfaces.

This control mechanism prevents unnecessary rodlet assembly and allows specifically a *single* layer of rodlets to form at the air:liquid boundaries or on fungal structures. This explains the apparent contradiction that while a non-agitated solution of MPG1 displays minimal increase in ThT florescence, rodlets form spontaneously at hydrophilic:hydrophobic interfaces without shaking (e.g. in nature or during AFM experiments). In the rodlet assembly assays, the role of the agitation is, on the one hand, to introduce a much larger hydrophilic:hydrophobic interface area into the sample than available in a static solution and on the other, to exchange assembled rodlets and monomer between interface and solution. This has the effect of maximising the conversion of soluble monomers into rodlets with the final ThT signal intensity scaling with the initial monomer concentration. Without agitation, a single layer of amphipathic rodlets occupying the air:liquid interface entirely will effectively shield the rest of the monomers from experiencing the hydrophilic:hydrophobic interface. Agitation allows all monomers in solution to eventually assemble into rodlets by continually re-exposing the air:liquid interface at the surface and possibly also inside the solution in the form of bubbles. When the interface is removed entirely, even agitation cannot induce rodlet assembly.

Apart from being critical for MPG1 rodlet assembly, hydrophilic:hydrophobic interfaces have been also shown to promote the assembly of disease-associated amyloids. In particular, for α-synuclein^36^, islet amyloid polypeptide^37^ and Aβ^38^, the presence of the hydrophilic:hydrophobic interface has been shown to result in a significant reduction of the lag phase and an increase in the total amount of aggregates formed (i.e. at the plateau). This interfacial effect on amyloidogenesis has been attributed to preferential adsorption of the surface-active peptides/proteins and aggregates at the interface which leads to increased surface concentration, promotion of conformational change and fibril elongation, thus drawing many parallels with the important role played by the interface in MPG1 assembly. However, unlike disease-associated amyloids, assembly of MPG1 rodlets cannot take place in the absence of the interface, even with the addition of seeds. In contrast, seeding greatly shortens the lag phase of disease-associated amyloid assembly and in the case for islet amyloid polypeptide, seeding has been shown to have very complex and even opposing effects on aggregation behaviour depending on the absence and presence of hydrophilic:hydrophobic interfaces^39^. These studies suggest that distinct pathways must exist for the assembly of disease-associated amyloid in solution and at an interface. However, it appears that hydrophobins have somehow evolved to specifically suppress the former pathway while enhancing the second such that the assembly is instantaneous (with no observable lag phase) once suitable interfaces are presented.

So what are some of the intrinsic structural features of class I hydrophobins that limit them to assemble *only* at hydrophobic:hydrophilic interfaces? Like other class I hydrophobins, the monomeric structure of MPG1 consists of a well-defined β-barrel core and the surface of the protein has large exposed hydrophobic regions and an amphipathic character, likely responsible for the propensity for hydrophobins to localise at an interface and to demonstrate surface activity. The kinetics suggest that these proteins can undergo a conformational change from an assembly-inhibited form in solution to an aggregation-competent form at the interface. We have previously demonstrated that rodlet assembly by the class I hydrophobin EAS is likely to be associated with conformational change^40^. The pattern of disulphide bonds in all hydrophobins, and the maintenance of these bonds in the rodlet structure, limits the conformational changes possible during self-assembly. In both EAS and MPG1, flexibility is observed in the loops that are present between the Cys3-Cys4 and Cys7-Cys8 disulphide tethers, thus identifying these regions as possible contributors to the intermolecular β-strand interactions that must occur upon amyloid formation. However, we have demonstrated that a large fraction of the EAS Cys3-Cys4 region can be removed without affecting the ability of the hydrophobin to fold and assemble into rodlets^14^. Indeed it is likely that the function of this highly flexible region is to reduce unwanted intermolecular association in solution, in the absence of an interface^41^. The Cys3-Cys4 region in MPG1 is also highly flexible and may play a similar role. Interestingly, hydrophobins which are known to exist as dimers or higher-order oligomers in solution appear to have relatively fewer flexible regions, at least in the small subset of hydrophobin structures that have been determined to date (e.g. HFPB1/II and DewA)^16^.

In EAS, the key amyloidogenic residues have been identified and are located in the Cys7-Cys8 region^40^. In the EAS rodlet model obtained using molecular docking, conformational change results in the Cys7-Cys8 region becoming more extended, thus exposing the amyloidogenic sequence contained within^40^. Our model suggests that MPG1 undergoes some level of conformational conversion between the form of the monomer in solution and an assembly-competent surface-associated structure. Consistent with the Cys7-Cys8 loop being a region important for this amyloidogenic switch, in the MPG1 structure in solution the Cys7-Cys8 loop shows a consistent, even though relatively minor, decrease in the hetero-NOE ratios and does not appear to make significant contact with the barrel core (Fig. 9B). In this way the Cys7-Cys8 region in the MPG1 solution structure more closely resembles EAS in the extended conformation observed in the rodlet model than in the monomer in solution (Fig. 9C). Further experiments are underway to directly investigate the role of the inter-cysteine regions in MPG1 rodlet formation.

This study demonstrates how structural features of hydrophobin proteins control self-assembly and allow the interplay between different classes of hydrophobins at growth and host interfaces to mediate function. The class II hydrophobin MHPI is highly surface-active and, when present with the class I hydrophobin MPG1, can suppress MPG1 rodlet assembly. Once formed, a layer of MPG1 rodlets may effectively enhance cutinase activity through localisation of the protein onto a surface or further activation of the protein through conformational changes. The partnering of these two hydrophobins could generate temporal and spatial control of rodlet formation and, as a consequence, cutinase activity, thereby providing a mechanism for the fungus to sense the leaf interface and to initiate the infection process through cutin hydrolysis. A further understanding of the molecular mechanism by which MPG1 and MHP1 assemble into the amphipathic layers and how Cut2 is activated will facilitate development of inhibitory strategies against rice blast infection.

## Materials and Methods

### Expression and purification of recombinant MPG1, MHP1 and Cut2

Synthetic genes encoding MPG1, MHP1 and cutinase2 (Cut2) from *M. oryzae* were sub-cloned into the pHUE expression vector to allow expression of fusion proteins with His_6_-ubiquitin at the N-termini^42^. His_6_-ubiquitin-MGP1 and His_6_-ubiquitin-MHP1 expression was performed using *E. coli* BL21 (DE3) and His_6_-ubiquitin-Cut2 expression was performed using Rosetta 2 (DE3) pLysS *E. coli*. Protein expression was induced with 0.5 mM IPTG for 3 h at 37 °C for His_6_-ubiquitin-MPG1 and for 4 h at 30 °C for His_6_-ubiquitin-MHP1 and His_6_-ubiquitin-Cut2.

All three proteins expressed in inclusion bodies were purified using Ni-NTA affinity chromatography under denaturing conditions as described^12^. After elution from Ni-NTA agarose beads, the proteins were oxidatively refolded via overnight dialysis. For His_6_-ubiquitin-MPG1 and His_6_-ubiquitin-MHP1, the refolding buffer contained 20 mM MES, 20 mM Tris, 5 mM reduced glutathione and 0.5 mM oxidized glutathione, pH 8. For His_6_-ubiquitin-Cut2, the refolding buffer contained 10 mM sodium acetate, 5 mM reduced glutathione and 0.5 mM oxidized glutathione, pH 5. After oxidative refolding, the proteins were subjected to enzymatic cleavage by deubiquitylating enzyme UBP41 and the His_6_-ubiquitin tag was removed using Ni-NTA agarose beads. The cleaved hydrophobins MPG1 and MHP1 were then further purified using rpHPLC and lyophilised before storage at −20 °C. Cut2 was purified by size exclusion chromatography (SEC) using a Superdex 75 10/300 GL (GE Healthcare) column and stored at 4 °C. The purity of the protein preparations was analysed by SDS-PAGE and the correct mass confirmed by mass spectrometry. Protein identity was confirmed using in-gel tryptic digestion followed by mass spectrometry. Folding of the proteins was confirmed using 1D ^1^H NMR spectroscopy.

### Size-exclusion chromatography coupled with Multi-angle Laser Light Scattering

A 200-μL sample of lyophilised MPG1 was prepared in 25 mM NaH_2_PO4, 150 mM NaCl, pH 7.2 (1.6 mg/mL) and was subjected to size-exclusion chromatography using a Superdex 75 10/300 GL column with an in-line MiniDawn Multiangle Light Scattering System (Wyatt Technology) connected to an AKTA FPLC machine (GE Life Sciences). MPG1 was eluted from the column at a flowrate of 0.5 mL/min in 25 mM NaH_2_PO4, 150 mM NaCl, pH 7.2 and light scattering, absorbance at 280 nm and refractive index of eluted sample were recorded. Molecular weight was calculated using the intensity of scattered light in combination with the change in refractive index using the ASTRA software (Wyatt Technology).

### NMR spectra acquisition

All NMR spectra were acquired on Bruker Avance III 600 or 800 MHz spectrometers equipped with 5-mm triple resonance TCI cryogenic probeheads (Bruker, Karlsruhe, Germany), processed using Topspin 2.1 (Bruker Biospin Ltd) and analysed with Sparky (T. D. Goddard and D. G. Kneller, University of California at San Francisco).

To optimize spectral quality and sample stability for MPG1 structure determination, one-dimensional (1D) ^1^H and two-dimensional (2D) ^1^H-^15^N-HSQC spectra were recorded under a range of conditions (including temperatures from 293–303 K and pH from 5.5–7.5). The optimal condition was 20 mM sodium phosphate, pH 5.5, containing 20 μM chloramphenicol (NMR buffer) at 298 K.

For MPG1 structure determination, samples (at a concentration of 300–400 μM) were prepared in NMR buffer in 5% or 100% D_2_O containing 20 μM sodium 2,2-dimethyl-2-silapentane-5-sulphonate (DSS) and placed into 3-mm or 5-mm Shigemi tubes (SHIGEMI Co. Japan). To minimize protein degradation, cOmplete, EDTA-free Protease Inhibitor Cocktail (Roche) was added to the NMR samples. Backbone and side-chain assignments were made using the following standard Bruker experiments: HNCACB, CBCA(CO)NH, CC(CO)NH-TOCSY, HNCO, HN(CA)CO and HBHA(CO)NH were recorded in H_2_O-based buffer while HCAN, HCA(CO)N, HCCH-TOCSY and H(C)CH-TOCSY were recorded in D_2_O-based buffer. NOE-derived distance restraints were obtained from ^13^C-edited NOESYs (aliphatic and aromatic versions) and a ^15^N-edited NOESY. Assignments were made using standard methodology. Φ and ψ-Angle restraints were predicted using TALOS+^43^.

### MPG1 structure calculations

NOESY spectra were peak picked manually and used as input into ARIA 1.2^44^ implemented in CNS (Version 1.1)^45^ together with 67 Φ and ψ angle constraints. Four disulfide bonds (C19-C77, C28-C51, C27-C71 and C78-C90) were incorporated in the calculations. The calculation protocol comprised a first cycle of 50 structures followed by seven cycles of 20 structures each and a final cycle of 500 structures. A total of 1315 unambiguous and 67 ambiguous restraints were identified by ARIA, and these were checked and corrected manually if necessary. The 50 lowest-energy structures from the last iteration were refined in a 9-Å shell of water molecules. The 20 conformers with the lowest value of Etot were visualized and analyzed using MOLMOL^46^, PROCHECK-NMR^47^ and PSVS^48^ and deposited into the PDB (accession code 2N4O).

### MPG1 relaxation analysis

Longitudinal (*T*_1_), transverse (*T*_2_) and heteronuclear NOE relaxation experiments were performed on ^15^N-labelled MPG1 (150 μM) in H_2_O-based buffer using standard Bruker pulse programs. The ^15^N-*T*_1_ inter-pulse time delays were 0.1, 0.15, 0.2, 0.3, 0.6, 0.8, 1, 1.4, 2.2 and 3 s and the *T*_2_ time delays were 17, 34, 51, 68, 85, 102, 136, 153, 170, 221 and 255 ms. Recycle delays of 4 s were used. Integrated peaks were fitted to two-parameter exponentials using the relaxation analysis module in SPARKY. ^1^H-^15^N Heteronuclear NOEs were calculated by taking the ratio of cross-peak intensities with and without proton saturation during relaxation delays.

### Contact angle measurements

For protein sitting-drop measurements, MPG1/MHP1 solution drops (10-μL) containing 100 μg/mL of MPG1 or MHP1 in MilliQ® water were incubated on OTS-coated silicon wafers inside a moist chamber (petri dish lined with a wet tissue) at room temperature for 50 min before contact angles were measured and/or images were taken. The drop shape was analysed using a Kruss DSA 10MK2 analyser (KRUSS, Hamburg, Germany) and accompanying software. Values given are the average from three drops.

For water contact angle measurements on MPG1/MHP1-coated surfaces, MPG1/MHP1 solution drops (50-μL) containing 100 μg/mL of MPG1 or MHP1 in MilliQ® water were incubated for 30 min before the bulk protein solution was carefully removed, and the surface was left to dry in air overnight. Water drops (10 μL) were dispensed at the centre of the protein-coated area in order to measure the water contact angle. The drop shape was analysed using a KSV CAM200 (Biolin Scientific, Stockholm, Sweden) and accompanying software.

### NMR titration studies

Lyophilised proteins were dissolved in titration buffer (25 mM NaH_2_PO_4_, 10% D_2_O, pH 6.5). Protein solutions were dialysed against titration buffer for 4 h and 10–20 μM of DSS were added to each sample before the titration. The final protein concentrations used in titration experiments were 80 μM for ^15^N-MPG1 and 400 μM for MHP1. ^1^H-^15^N HSQC spectra were recorded after sequential additions of unlabelled MHP1 to obtain molar ratios of [MHP1:^15^N-MPG1] of [0.2:1], [0.4:1], [0.6:1], [0.8:1], [1:1], [1.2:1] and [2.5:1]. After each addition, the solutions were mixed and equilibrated for 5 min before spectra were recorded.

### Transmission electron microscopy

A small volume (2.5 μL) of MPG1 solution in water (50 μg/mL) was placed onto the surface of a carbon-coated formvar film on a copper grid (ProSciTech GSCu200C) and left to dry overnight at 37 °C. The sample was stained by floating the coated-surface downwards on a drop of 2% uranyl acetate solution and then removing excess stain by wicking with filter paper. The grid was examined in a Philips CM12 electron microscope operating at 120 kV and images recorded using a Morada CCD camera.

### Atomic force microscopy

A small volume (20 μL) of MPG1 solution in water (5 μg/mL) was placed onto a freshly cleaved HOPG surface and allowed to dry overnight at room temperature free from dust. The sample was then imaged using a Veeco Nanoscope IIIa (Veeco, USA) operating in tapping mode under ambient conditions. A silicon cantilever (BudgetSensors, Innovative Solutions Bulgaria) with a free resonance frequency of 300 kHz, spring constant of ~40 N/m and tip radius under 10 nm was used for sample imaging.

### Hydrophobin rodlet assembly assay

Thioflavin-T (ThT) fluorescence assays were used to monitor rodlet formation by hydrophobins. Rodlets, but not soluble hydrophobins, have an underlying cross-β structure which binds the amyloid-specific dye ThT and results in increased fluorescence. Rodlet formation was induced by double orbital shaking at 300–700 rpm and at 25–50 °C in a Costar 96-well fluorescence plate (Corning) inside a POLARstar Omega microplate reader (BMG Labtech). Unless otherwise stated, protein solutions (100 μL, 25 μg/mL) were prepared in 25 mM NaH_2_PO_4_, pH 7.2 containing 20–40 μM ThT in the presence or absence of additives. For pH assays, experiments were performed in 20 mM Tris or sodium acetate to achieve a pH of 3 or 7, respectively. The exact condition (including additives) used is detailed in the legend or results section. Samples in triplicate were excited at 440 nm and fluorescence emission was measured at 480 nm every 60 s.

### Analysis of assembly mechanism

The proposed mechanism of aggregation proceeds as follows: A constant number of growth competent seeds, *P*_0_, elongates by addition of monomers, *m*, in a mechanism that can saturate at high monomer concentrations. The differential equation for the rate of formation of the total aggregate mass concentration, *M*(*t*), is then given by:


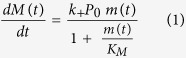


where *k*_+_ is the elongation rate constant and *K*_*M*_ is the Michaelis constant of elongation, which gives the monomer concentration at which saturation effects become important. In the low monomer concentration regime, i.e. the unsaturated regime, the rate of aggregate production becomes 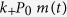, linearly dependent on monomer concentration. In the high monomer concentration regime, i.e. the fully saturated regime, the rate of aggregate production is given by 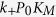 which is independent of the monomer concentration.

This differential equation can be solved to yield the time evolution of the total aggregate mass concentration, as described in the Supplementary Information, which is then fitted to the experimental data as shown in Fig. 8. Note that the two constants *k*_+_ and *P*_0_ appear only as the product *k*_+_*P*_0_, therefore their individual values cannot be determined. There are hence only 2 independent fitting parameters, *k*_+_*P*_0_ and *K*_*M*_, which were fitted in a global manner to all monomer concentrations.

### Circular dichroism (CD) spectropolarimetry

Far-UV CD spectra from 190 to 250 nm were recorded on a solution of Cut2 (18 μM) in 10 mM NaH_2_PO_4,_ pH 8 in a Jasco 720 spectropolarimeter at 20 °C, using a 1-mm path length quartz cuvette. Data were baseline-corrected with buffer alone.

### Esterase assay

Cutinase is an enzyme capable of hydrolysing ester bonds. The activity of Cut2 was determined using *p*-nitrophenyl butyrate as substrate (Sigma) and monitoring the release of *p*-nitrophenol at 400 nm using a POLARstar Omega microplate reader (BMG Labtech). Solutions containing Cut2 (100 μL, 40–50 nM) in 25 mM NaH_2_PO4, pH 7.2 were incubated at room temperature for 30–60 minutes in the absence and presence of proteins and additives before 100 μL of 1 mM *p*-nitrophenyl butyrate was added to the reaction and the absorbance at 400 nm was immediately monitored for up to 4 hours. Negative control samples containing all components apart from Cut2 were also measured. All measurements were performed in triplicates. For determination of enzyme kinetic parameters (K_m_ and K_cat_/K_m_) using a Lineweaver-Burke plot, the esterase assay was performed with 12.5 nM Cut2 over a concentration of *p*-nitrophenyl butyrate ranging from 0–8 mM.

### BioLayer Interferometery (BLI)

Biotinylation of MPG1 was performed on a BLItz (Pall Life Sciences, Melbourne, Australia) using EZ-link^®^ NHS-PEG_4_-biotin according to the manufacturer’s protocol (PIERCE, Rockford, IL, USA). After equilibrating the streptavidin biosensor in PBS with 0.005% Tween-20, an initial baseline was collected and immobilisation of biotinylated MPG1 (5 μg/mL) was achieved by flowing the protein solution over the biosensor for 2 min. MHP1 (0.25 mg/mL and 0.6 mg/mL in PBS with 0.005% Tween-20) were then flowed onto the immobilised biotinylated MPG1 (association step) followed by buffer (dissociation step). The binding kinetics were analysed using the advanced kinetic function within the BLItz software.

### Cutinase binding and activation assay

Protein solution (80 μL of 5 μg/mL MPG1, MHP1, lysozyme or BSA in MilliQ water) was pipetted onto a freshly cleaved HOPG surface. Control surface was prepared by pipetting 80 μL of water. Samples were then allowed to dry at room temperature for 20 h.

A 50-μL solution of Cut2 (50 nM) in 25 mM NaH_2_PO_4_, pH 7.2 (buffer) was pipetted onto each of the dried protein surfaces and incubated at room temperature for 30 min, after which the Cut2 solution was pipetted off. Surfaces were washed five times by pipetting 50 μL of buffer onto the surface, incubating for 1 min and then removing by pipetting. Cut2 activity was determined at room temperature by addition of a 50-μL drop of 1 mM *p*-nitrophenyl butyrate in buffer onto the surfaces. At various time points (5, 15 and 30 min) after substrate addition, 5 μL from each sample was pipetted off for absorbance measurement at 400 nm using a Nanodrop™ spectrophotometer (Thermoscientific).

## Additional Information

**How to cite this article**: Pham, C. L. L. *et al.* Self-assembly of MPG1, a hydrophobin protein from the rice blast fungus that forms functional amyloid coatings, occurs by a surface-driven mechanism. *Sci. Rep.* **6**, 25288; doi: 10.1038/srep25288 (2016).

## Supplementary Material

Supplementary Information
