# Supplementary material for: Self-assembly of MPG1, a hydrophobin protein from the rice blast fungus that forms functional amyloid coatings, occurs by a surface-driven mechanism
Source: Sci Rep. 2016 May 4;6:25288. doi: 10.1038/srep25288 (PMC4855151; doi:10.1038/srep25288)
Supplement: Supplementary Information [file srep25288-s1.pdf]

Supporting Information: Self-assembly of MPG1, a hydrophobin protein from the rice blast fungus that forms functional amyloid coatings, occurs by a surface-driven mechanism.

Chi L. L. Pham[1], Anthony Rey[2], Victor Lo[1], Margaux Soules[1], Qin Ren[1], Georg Meisl[3], Tuomas P. J. Knowles[3], Ann H. Kwan[2*], and Margaret Sunde[1*]

[1]Discipline of Pharmacology, School of Medical Sciences and [2]School of Molecular Bioscience, University of Sydney, NSW 2006, Australia [3]Department of Chemistry, University of Cambridge, Lensfield Road, Cambridge CB2 1EW, UK.

Modeling of aggregation kinetics

Deriving integrated rate laws

In general the aggregation reaction is described by three quantities, the free monomer concentration $m(t)$, the fibril mass concentration $M(t)$ and the number concentration of growth competent ends $P(t)$. Nucleation predominantly increase P whereas growth processes are responsible for an increase in M[1, 2]. In the present system, both the nucleation and the growth processes were found to be independent of the bulk monomer concentration m, until most of the free monomer is depleted, shortly before completion of the aggregation. Moreover the lack of curvature in the kinetics suggested that $P(t)$ is in fact time-independent (see below for details of this argument). Therefore the mechanism we propose proceeds via a constant number of growth competent ends, P_0, and an elongation process that is monomer independent at high monomer concentrations[3]. The differential equations describing the aggregation reaction are then given by:

$$\frac{dP}{dt} = 0 \tag{1}$$

and

$$\frac{dM}{dt} = k_+ P(t) \frac{m(t)}{1 + m(t)/K_M} \tag{2}$$

where k_+ is the elongation rate constant and K_M in the denominator on the right hand side captures the saturation propensity of the elongation rate (more on this below).

We use the initial conditions $P(t) = P_0$ and $M(t = 0) = 0$. As stated in the main text, the initial nucleation event is very fast and the number of growth competent nuclei does not change after this initial nucleation event, which is captured by $P(t) = P_0$, where P_0 is the amount of nuclei present effectively instantly at the beginning of the experiment. As the increase in fluorescence due to this initial nucleation event is negligibly small, we assume here that the amount of aggregate material created during initial nucleation is also negligible, hence we set $M(t = 0) = 0$.

Imposing mass conservation as $m_{\text{tot}} = M(t) + m(t)$ we obtain

$$\frac{dM}{dt} = k_+ P_0 \frac{m_{\text{tot}} - M(t)}{1 + (m_{\text{tot}} - M(t))/K_M} \tag{3}$$

which can be solved to give

$$M(t) = m_{\mathrm{tot}} - K_M W \left[\frac{m_{\mathrm{tot}} e^{\frac{m_{\mathrm{tot}}}{K_M} - k_+ P_0 t}}{K_M} \right] \tag{4}$$

where $W[x]$ is the Lambert W function, defined by $x = W[x]e^{W[x]}$.

This solution for $M(t)$ was used to fit the experimental data in the main paper. Note that the constants k_+ and P_0 only appear as a product hence they can not be determined individually. Note that this constant, k_+P_0, should only be interpreted as an effective growth rate in the context of this specific setup: in addition to the intrinsic rate of addition of a monomer to a growing fibril, it is also dependent on other system parameters, such as how quickly monomer and fibrils are exchanged between interface and bulk, which are likely influenced by the shaking speed and other setup specific factors.

Lack of curvature implies constant number of growth competent ends

The claim is that an increase of the number of growth competent ends always leads to positive curvature at early times. At early times the free monomer concentration remains approximately constant (i.e. monomer depletion is negligible). Therefore, very generally, the rate of increase of mass is given by:

$$\frac{dM}{dt} = kP(t) \tag{5}$$

where k is the rate of elongation (monomer independent in this early time limit) and of course $k > 0$. This is true in general assuming that the increase in mass from nucleation processes is negligible. Differentiating this expression once gives the curvature of the aggregation curves:

$$\frac{d^2M}{dt^2} = k\frac{dP}{dt} \tag{6}$$

Therefore if the number of growth competent site increases, i.e. $\frac{dP}{dt} > 0$, there is positive curvature. Equally, if the number of growth competent sites is constant, $M(t)$ increases linearly already at early times.

Origins for saturation effects in elongation

The elongation reaction is monomer independent for most of the reaction, however, eventually the monomer concentration becomes very low and the elongation rate has to become monomer dependent in order for the aggregation reaction to stop once all monomer is used up (i.e. when the free monomer concentration is equal to the solubility). One way of achieving this is by explicitly treating the two steps of elongation by monomer addition: initially monomers have to attach to fibril ends, the rate of this process is dependent on the monomer concentration, and then they will have to rearrange into the fibrillar structure, the rate of this process is monomer independent. If the monomer concentration is high, corresponding to a fast attachment step, the rearrangement becomes rate limiting and the overall reaction is independent of the monomer concentration. The kinetics of this process can be captured by Michaelis Menten kinetics and this was used in equation (3) above, where the rate of elongation (equivalent to the rate of increase of M in this case) was given by

$$r = k_+ P_0 \frac{m(t)}{1 + (m(t))/K_M} \tag{7}$$

The Michaelis Menten reaction involves three species: free monomer, free growth competent ends and a monomer-end complex, which we will refer to as the intermediate. Now define the rate constant of formation of intermediate from free monomers and free ends as k_1 and its reverse as k_2 and the rate of formation of products (which in this case is a slightly longer fibril with a growth competent free end) as k_3. By assuming steady state of the intermediate, we can derive the usual Michaelis Menten kinetics, where we identify $K_M = (k_2 + k_3)/k_1$ and $k_+ = k_3/K_M$.

An alternative explanation for the observed independence of the elongation rate on the bulk monomer concentration could be the saturation of the air-water interface with monomer. In the simplest model, the amount of monomer bound to the surface is described by the Langmuir isotherm:

$$\phi = \frac{am}{1 + am} \tag{8}$$

where ϕ is the fraction of the surface that is occupied, a is a constant and m is the bulk monomer concentration. Note that this is the same functional form as obtained above, so mathematically these two descriptions are equivalent and a kinetic analysis can therefore give no insights into which of those two mechanisms is the relevant one in the current system. Although they are mathematically equivalent, the interpretation of the constants in the 2 models differs: K_M, or $1/a$ gives the concentration at which the fibril ends saturated in the Michaelis Menten model and the concentration at which the surface sites saturate in the Langmuir model.

References

[1] Samuel I A Cohen, Michele Vendruscolo, Mark E Welland, Christopher M Dobson, Eugene M Terentjev, and Tuomas P J Knowles. Nucleated polymerization with secondary pathways. i. time evolution of the principal moments. *J Chem Phys*, 135(6):065105, Aug 2011.

[2] Tuomas P J Knowles, Christopher A Waudby, Glyn L Devlin, Samuel I A Cohen, Adriano Aguzzi, Michele Vendruscolo, Eugene M Terentjev, Mark E Welland, and Christopher M Dobson. An analytical solution to the kinetics of breakable filament assembly. *Science*, 326(5959):1533–1537, Dec 2009.

[3] Georg Meisl, Julius B. Kirkegaard, Paolo Arosio, Thomas T. C. Michaels, Michele Vendruscolo, Christopher M. Dobson, Sara Linse, and Tuomas P. J. Knowles. Molecular mechanisms of protein aggregation from global fitting of kinetic models. *Nature Protocols*, 11(2):252–272, 2016.
